# A comprehensive survey on the biomedical signal processing methods for the detection of COVID-19

**DOI:** 10.1016/j.amsu.2022.103519

**Published:** 2022-04-01

**Authors:** Satyajit Anand, Vikrant Sharma, Rajeev Pourush, Sandeep Jaiswal

**Affiliations:** a Electronics and Communication Engineering, Mody University of Science and Technology, India; b Mechanical Engineering, Mody University of Science and Technology, India; c Biomedical Engineering, Mody University of Science and Technology, India

**Keywords:** Signal processing, COVID 19, Artificial intelligence, Audio, *Speech*

## Abstract

The novel coronavirus, renamed SARS-CoV-2 and most commonly referred to as COVID-19, has infected nearly 44.83 million people in 224 countries and has been designated SARS-CoV-2. In this study, we used ‘web of Science’, ‘Scopus’ and ‘goggle scholar’ with the keywords of “SARS-CoV-2 detection” or “coronavirus 2019 detection” or “COVID 2019 detection” or “COVID 19 detection” “corona virus techniques for detection of COVID-19”, “audio techniques for detection of COVID-19”, “speech techniques for detection of COVID-19”, for period of 2019–2021. Some COVID-19 instances have an impact on speech production, which suggests that researchers should look for signs of disease detection in speech utilising audio and speech recognition signals from humans to better understand the condition. It is presented in this review that an overview of human audio signals is presented using an AI (Artificial Intelligence) model to diagnose, spread awareness, and monitor COVID-19, employing bio and non-obtrusive signals that communicated human speech and non-speech audio information is presented. Development of accurate and rapid screening techniques that permit testing at a reasonable cost is critical in the current COVID-19 pandemic crisis, according to the World Health Organization. In this context, certain existing investigations have shown potential in the detection of COVID 19 diagnostic signals from relevant auditory noises, which is a promising development. According to authors, it is not a single “perfect” COVID-19 test that is required, but rather a combination of rapid and affordable tests, non-clinic pre-screening tools, and tools from a variety of supply chains and technologies that will allow us to safely return to our normal lives while we await the completion of the hassle free COVID-19 vaccination process for all ages. This review was able to gather information on biomedical signal processing in the detection of speech, coughing sounds, and breathing signals for the purpose of diagnosing and screening the COVID-19 virus.

## Highlights

• This is a comprehensive review of published work for the detection of COVID-19.
• Previously conducted studies on audio, voice, cough sound, breathing and signal processing methods in order to address COVID-19-related health conditions.
• Analyzing and diagnosing COVID-19 using audio, speech and Signal Processing.
• Diagnosing and Screening of COVID-19 are studied using Machine Learning, Artificial Intelligence and Deep Learning.
• An overall these Signal Processing, Machine Learning, Artificial Intelligence and Deep Learning techniques were seen to have satisfactory results for the detection of COVID-19.

## 1 Introduction

Biomedical signals are gathered from the body at the cellular, organ, and molecular levels. There are several types of biomedical signal processing, including EEG (electroencephalogram), which infers the electrical activity of the brain; ECG (electrocardiogram), which infers the electrical activity of the heart; EMG (electromyogram), which infers the electrical activity of muscle noise signals; electroretinogram and electroneurogram, which infer the electrical activity of the eye; and so on. Biomedical signals are initially utilized for diagnosing or detecting specific physiological and pathological states. Additionally, these types of signals are used in the medical care business for the analysis of biological systems [1]. This aims for signal de-noising, feature extraction, exact recognition of signal model, dimensionality reduction for dysfunction or decisive function, and prediction of future pathological and functional occurrences by implementing AI (Artificial Intelligence) models. According to this article, it demonstrates how biomedical signals are used in the health care industry, as well as detecting COVID-19. In particular, the major contribution of this review study is on the analysis of COVID-19 and how it can be used to detect symptoms using a variety of signal processing approaches. The information for this study was gathered through journals and published papers, and it was completed by the year 2021. Many efforts are made to collect data from COVID-19 patients in order to detect the virus. The bulk of COVID-19 symptoms are related to the respiratory working system, which has a significant impact on human speech output [2]. This study paper presents a summary of signals processing using artificial intelligence to diagnose, screen monitor, and raise awareness about COVID-19. This study goes on to provide a more in-depth discussion. This paper will examine the present state of research on this subject. Following are the research questions that will be examined:

RQ1: Which Biomedical Signal Processing methods are used for the detection of COVID-19?

RQ2: Which Techniques are used to detect audio and cough sound?

RQ3: Which Artificial Intelligence Techniques are used in the same field?

## 2 Methods

The goal of this study aimed to know about various biomedical signal processing methods and AI techniques in the field of COVID-19. The references which are included in this overview, we used ‘web of Science’, ‘Scopus’ and ‘goggle scholar’ with the keywords of “SARS-CoV-2 detection” or “coronavirus 2019 detection” or “COVID 2019 detection” or “COVID 19 detection” “corona virus techniques for detection of COVID-19”, “audio techniques for detection of COVID-19”, “speech techniques for detection of COVID-19”, for period of 2019–2021. The study is based on data that is available to the public. This study doesn't use any patient data or human subjects. So it doesn't require an ethics committee review [3].

## 3 COVID-19 diagnosis by signal processing of audio, speech, language

Regular symptoms of COVID-19 are dry cough, fever, and fatigue, and also the severe symptoms of COVID-19 are loss of appetite, shortness of breath, persistent pressure or pain in the chest, confusion and temperature raise above 38° Celsius. Supervising and screening the population on the development of pandemic situation is compulsory. Among the alternative methods for detecting COVID-19, evaluation of human audio signals has some of benefits: It is easy for obtaining, non-intrusive; assessment and recording can be done immediately. An open research question is whether the human audio signal offers sufficient ‘markers’ for COVID-19, which results in perfect performance of classification so, that COVID-19 explained in detail apart from the other types of respiratory diseases. The heavy droplets are produced when infected person sneezes or coughs which transmits the virus causing COVID-19. While talking or breathing closely to someone, there is 100% chance of transmitting COVID virus form one person to other. With the close consideration of these transmitting symptoms and factors, each and every individual person towards with health care professional is must aware about to stop the spread of this virus [4].

It is most important to have an easy tool for diagnosing, screening and supervising the virus and its proliferation. An automatic method is used for detecting and monitoring the presence of COVID-19 or its symptoms are developed using AI (artificial intelligence) based approaches [5]. Many AI techniques using speech and other audio models having many opportunities in this space [6]. For scaling up the detection of COVID-19 virus, this section provides different applications and algorithms using the audio processing signals for diagnosing and screening of COVID-19. The topic ‘corona virus’, ‘audio’, ‘speech’ techniques for detection of COVID-19 discussed about the processing and capturing the speech and other speech related human data for diagnosing and screening of COVID-19 [7].

### 3.1 Cough detection

Cough is the one among the eminent symptoms of COVID-19; it creates interests for knowing the techniques which is used for detecting human cough and discerning it form the other same sounds like speech and laughter [8]. CSI (Cough signature identification) helps for differentiating the cough sounds and identifies reasons of cough as which bacteria or virus is affected [9]. The followed studies explained in detail about the audio features used for detecting cough sounds: The audio based sensing techniques can sustain the physical distance by calculating the frequency, intensity and features of COVID-19 cough [10]. Moreover, the possibility of accumulating coughs directly from patients is less in the short term. Additionally [11], introducing a novel database named as NoCoCoDa which includes COVID-19 cough events, is attained via public interviews in media with COVID-19 patients, as the temporary solution. After manually segmenting interviews, a total of 73 cough events are individually extracted and CPA (Cough Phase Annotation) was implemented [12]. The NoCoCoDa is structured and used for rapid investigation and algorithm development, which is further used for applying the more extensible datasets and possibly in real time applications. Similarly [13], investigating the use of symbolical regression quantification measurements for detecting COVID-19 automatically towards the cough sounds of healthy and sick individuals. The performance evaluated the symbolical dynamic measurements which are highly efficient at discerning healthy and sick coughs. The model attained the MC (Mean Classification) evaluation performance of 99% and 97% and F1 score with 89% and 91% after optimizing sustainable vowels and coughs respectively.

Likewise [14] presenting the detailed automatic system for detecting COVID-19 from the recordings of cough, submitted by PANACEA team. This study established various systems based on developing signal processing and ML (machine learning) methods. This system implemented a TECCs (Teager energy operator cepstral coefficients) as the front end and LightGBM (Light gradient Boosting Machine) as the back end. The AUC attains a test set with 76.31% which related to a 10% enhancement over the official base line of this system. Same type of the study has been conducted by Ref. [15], demonstrates that entreated cough sounds are gathered over a phone call, and analysed by AI models, indicates COVID-19 status of (AUC 0.72, *t*-test, p < 0.01, 95% CI of 061–0.83) statistically. This technical tool increases the testing capability of health care system by 43% at the disease incidence of 5%, without adding extra supplies, physical infrastructure or trained personnel. In the same way [16] trailed the enhancement status of an audio based cough monitor and detecting system for briefly describing the history of unbiased cough detection and then illustrating the cough noise or sound producing principle. The potential end points of cough studies, includes cough frequency, acoustic properties of cough noise or sound and intensity of coughing were analysed in this paper.

### 3.2 Voice detection

Voice based models are initiated for discerning COVID-19 positive cases form health controls regulates in Ref. [17]. The productiveness of this models are measured on crowd sourced data set and high lights the highest potentials for establishing an initial stage for screening tool based on voice signals for diagnosing the disease. In addition to voice analysing of this work considers fusion strategies for combining voice and reporting symptoms for getting better results. It does not incorporate other types of noises or sounds as coughing with less voice and breathing are the limitations of the study. Furthermore, this study investigating the impacts of the disease through voice, analysing the voice before and after infection. According to this study [18], the detection of COVID-19 through voice by pre-screening method which leads to automatic identification of COVID-19 using analysis of TFR (time frequency representations) with same performance [19]. presenting dataset for cough, voice, audio recording for breathing gathered form individuals infected by SARS-CoV-2 virus, and also the non-infected subjects as large scale crowd sourced operation. The study explained about the initial results for detecting COVID-19 form coughing sound patterns using the basic acoustic features sets, deep audio embeddings, and wavelet scattering features are obtained from low stage of feature representations OpenL3 and VGGish. This models attained accuracy rate about 88.52%, specificity about 90.87% and sensitivity about 88.75% which confirms the applicable audio segmentation signatures for detecting COVID-19. If voices are changes due to infection, then it is correlated to combine acoustic measurements such as formant characteristics, basic frequency, voice perturbations such as shimmer and jitter for different vowel sounds and HNR. Hence, analysing the voice used for prognosis and scanning of COVID-19 infection. It is based on the findings of [20], an application is developed in mobile for analysing the human voice to identify COVID-19 real time symptoms for remedial measurements and necessary action.

### 3.3 Speech detection

**Fig. 1:**
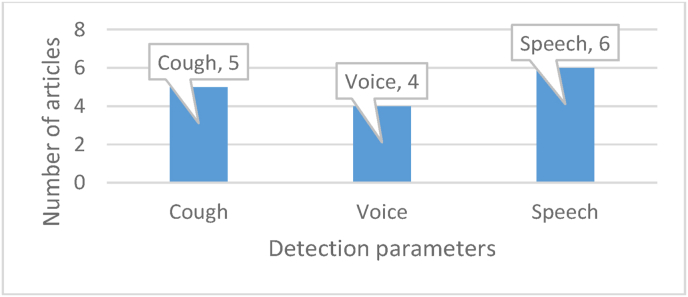
Number of articles presented for analysing the cough, voice and speech signal parameters for the detection of COVID 19.

The perception of speech signal is controlled by three factors: pitch, timbre and volume. The volume of speech is measured by sound intensity and its related amplitude of the signal. Pitch is basic frequency of speech signal and it measures how a particular objects receives the sound. Timbre is decided by sound harmonics and related to the components of frequency of signal spectrum [21]. presented general adversarial network DL (deep learning) for immediate identification of COVID-19 through speech signals. This system includes two levels, classification and pre-processing. This work implemented LMS (least mean square) filter for removing the sounds or noises or artifacts form inputted speech signals. After eliminating noises, the GANC (Generative Adversarial network classification) method for analysing the FCC (Frequency Cpestral Coefficients) and classifying the non- COVID-19 signals and COVID-19 signals. The results explained a more eminent correlation of MFCCs with many COVID-19 breathing and cough noises, the sounds must be powerful and clear to differentiate non- COVID-19 and COVID-19 models. Similarly [22] suggested a signal processing framework and speech modelling for detecting and tracking COVID-19 through symptomatic and asymptomatic stages. This model is based on complication of neuromotor co-ordination over speech subsystems concerned in phonation, respiration and articulation and driven by distinct nature of COVID-19 which includes lower diaphragm, bronchial and lower tracheal vs. upper area like pharyngeal, oral, laryngeal and nasal of respiratory tract redness and also by the developing proof of virus'neuro logical expressions. Validation is necessary for huge amount of datasets and for addressing the confounding influences like unbalanced data quantities, various recording conditions, and changing essential vocal status form previous and post time of recording. A technique for identifying COVID-19 symptoms before it became worse, so the person must be quarantined, tested and offered with medical support early as possible [23]. The Cepstral features are analysed for speech recognition and optimizing the conversing scale in frequency domain, frequency range of filtering banks of bio inspiring methods for achieving better COVID-19 identification has been evaluated by Ref. [24]. This technique easily diagnose the initial stages of virus conditions in patients without visiting hospitals and without any help of medical staff, it provides automatic detection of virus [25]. used a technical approach same as the speech recognition. Every statement is represented as super vectors of short term MFB (Mel filter bank) features for every fundamentals [26]. illustrated about the significance of speech signal processing in the derivation of MFCC of non- COVID-19 and COVID-19 samples and locates the relation using PCC (Pearson's Correlation Coefficients). Hence, AI can be used for diagnosing and early detection of COVID-19 through breath, cough, voices and speech (see Fig. 1).

## 4 Biomedical signal processing

The biomedical signals includes various types of artifacts with external or internal intruding sounds. These artifacts are removed by implementing signal de-nosing methods for filtering out the highest artifacts and noises. The biomedical signal analysing and processing are developed in three main steps:

• De-noising/pre processing
• Dimensionality reduction/feature extraction and
• Classification/detection.

### 4.1 Denoising or pre-processing

The main aim for pre-processing is for simplifying succeeded procedures without losing the related details and information and for enhancing the signal quality by increasing the SNR (signal to noise ratio). Transformations and filters like PCA, KPCA, MSPCA and ICA are most often used for pre-processing. Researchers employing these techniques for eliminating or reducing the unrelated signal elements by converting the signals. COVID-19 deaths patients also have cardiovascular disease. Thus, the ECG signal de-noising is important for obtaining useful signals and in correspondence to Ref. [27] use DWT (discrete wavelet transform) for processing ECG signals. Due to the tightness of ECG signal processing, it has great impacts on the signal, so Daubechies wavelet is more relatable. In order to get uses of ECG signals, the study de-noised the low frequency or high frequency sounds. Additionally, a complete ECG is the collection of the long continuous time series [28]. If it is straight inputted into the neural network classification, it will be enhance the difficulty of network classification and it is useful for the extraction of perfect feature details and information. Further [29] develops HDSD (Hybrid deep speech de-noising technique which do not relied on available clean speech signals. A fully conventional neural network has been skilled by using two noisy accomplishments of same voice speech signals; one has been used for inputting and other as the outputted results of the network. Many pre-processing techniques are accessible in literature, includes techniques for removing artifacts in recordings of ECG, the determining efficient of frequency bands of EEG signals, identifying related electrodes [30,31]. These techniques are attempting to remove OA (ocular artifacts) for obtaining a high quality EEG to eliminate artifacts in recordings of EEG. Similarly [32], aims for improving the accurate values of BCI (brain computer Interface) in signal classification of EEG by homologous chromosome pairs which compared to the symmetrical channels in which equal regions of homologous chromosomes which are related with the sample of ranges [33].

### 4.2 Feature extraction

Feature extraction is one among the critical steps in biomedical signal analysis. Thus, biomedical signals are composed with various data points, and information are retrieved using various feature extraction techniques [34]. These information and distinctive parameters describes the attitude of signal waveform with accurate action. The biomedical signal patterns are represented by amplitudes and frequencies [35]. These features are extracted by using various types of feature extraction methods, is the another mile stone for simplifying the signal processing steps for classification. The biomedical signals are decayed using the TF (time frequency) methods which detect changes in frequency and time. It is most important to handle small number of values which characterize the proper features of signals for accomplishing best performance [36]. Features are basically gathered into a feature vector by converting signals into related feature vector known as feature extraction. Unique features of the signal are examined by signal classification framework and depends upon those unique features, and the class of signal is also determined [37]. TF techniques like WVT (Wigner-Ville transform), STFT (short time Fourier transform) and WT (wavelet transform), WPT (wavelet packet transform), DWT (discrete wavelet transform), TQWT (tunable Q-factor wavelet transform), EMD (Empirical mode decomposition), ensemble EMD and DTCWT (Dual tree comple wavelet transform) and decomposition signals in frequency domain and time. A Capsule network is refers to as “CT-CAPS”, is presented in Ref. [38] for automated extraction of features in chest CT scans. These features are extracted from layer which lies before the final capsule layer and influenced for differentiating non- COVID-19 and COVID-19 cases. Similarly [39], finished feature extraction form the data set of 3 classes of COVID-19, pneumonia and normal lung images are created, with the each class of 364 pictures with DL methods like VGG19, Alexnet, ResNet and GoogleNet. For the feature selection, the two algorithms named PSO and GWA are used. After feature selection, the classification is done by using SVM. Hence, the accuracy of this approach are 99.38% [40].

Analysis is made through the crypt, iris pigment spot and wolflin nodules biological features by Ref. [41]. The features are segmented individually in the form of rectangle. The SURF, BRISK, MinEigen, FAST, Harris and MSER are elicited form crypt, pigment spot of iris region and wolflin nodule [42].The result of pigment spot of iris region, crypt and wolflin nodule are obtained by using statistical analysis. The total inputs are divided into trained and untrained category as 60% and 40%. The test and train method used for feature matching performance. The authenticated threshold level of crypt, wolflin nodule and pigment spot are as 2, 0, and 3 respectively [43]. improves the speech recognition system using various features of extracting methods. The work emphasising the pre-process of related audio samples where noises from speech samples has been removed using filters. Then, DWT (discrete Wavelet transforms), MFCC (Mel Frequency Cepstral Coefficients), ZCR (Zero crossing rate), pitch and energy are used for extracting the features. In feature selecting stages GFA (Global feature algorithm) is used for removing unwanted details from features and for identifying the emotions form the ML methods. These algorithms validating global emotions like sad, happiness, anger and neutral [44]. developing a new toolbox called SPAC for simulating and extracting speech attributes. The vibrated signals are disintegrated into IMFs (Intrinsic mode functions) by CEEMD algorithm because it has good adaption for extracting non stable signals form features. Then [45], it improved as LDWPSO algorithm which is initiated for solving the problem where the selection of smooth factor in PNN. Hence, the diagnosing COVID-19 using LDWPSO-PNN. The proposed method measure experimented datasets. The results indicates that the methods are feature selected of vibrating signals and distinguishing them efficiently [46]. A parallel framework based on MPI for the large database dataset for extracting power spectrum features of EEG signals for improving the speed of BCI [47].

### 4.3 Classification

A ML (machine learning) based on COVID-19 cough classification which will discriminating COVID-19 negative coughs from COVID-19 positive coughs these are recorded using smart phones [48]. The study identifies the best performing classification Resnet 50 is able to discriminating coughs of COVID-19 on the dataset of Coswara. Hence, the result of AUC is 0.7% and the result of Coswara dataset with 0.94. The best feature selection method in recently is SFS (Sequential forward selection). Furthermore, the better performance is acquired with the large number of datasets of MFCCs, it differentiates non- COVID-19 and COVID-19 coughs [49,50]. develops a new facial mask condition identifies methods by combination of pictures by SRCNet (Super resolution and classification networks), calculates three classification problems on unrestrained 2D facial images [51]. The proposed algorithm includes four steps: facial detection, and cropping, image pre-processing, facemask wearing identification and image super resolution. Finally, the SRCNet attained 98.70% of accuracy and performs end to end classification methods using DL techniques with super resolution of images over 1.5%. The results indicates that the proposed SRCNet acquires highest accurate values [52]. identifies different coughs sounds for altering real time life environments (see Table 1).

**Table 1:** Table representing the survey on existing methods of biomedical signal processing in the detection of COVID 19.

Method	Description	Pros.	Cons.
A novel algorithm of signal processing with Sparsity filter for eliminating noises in frequency^59^	An low cost global environment sensor	This proposed system performs the respiration rate of 98.98% in monitoring.	Towards with advanced sensors this barometric sensors can extended with its quality of respiratory monitoring also in sleep.
WIFI-COVID^60^	This system monitors RR (respiration rates) of patients who are COVID-19 positive with available source of home WiFi.	A new method is created for the extraction of RRs form CSI with high resolution spectrogram.	These RR is only used the patients under self-isolation and self-quarantine surroundings.
New frame work with five components^76^ Gathering and uploading the symptom data Isolation/quarantine centre Analysis centre for data Cloud infrastructure and health physicians.	Quickly identifying the Corona virus with real time data with eight ML algorithms such as NN, SVM, K-NN, NB, DS, DT ZeroR and OneR are conducted to test COVID-19.	Only five algorithms attained results with best accurate values of 90% for identifying the test results of COVID-19.	The result explains that except DS, ZeroR and OneR algorithm are not attained best accuracy results
New methods called IoT protocols, R-MAC and TS-MAC and ultra-low level latency with 1 ms is attained^61^	This work proposes an integrated communication networks using wireless physiological signals for processing LSTM based recognition of wearable devices.	This system proposed emotion recognition paradigm which supports and assists students and health care professional with DL method to stop the outbreak of COVID-19.	This paper can be extended in future to end to end communication and visual aids for supporting distance learning and incorporating the services.
A two tiered system is created using wearable devices to consumers^80^	This paper analysed physiological and activity data with detected emotions and physiological alterations as symptoms of COVID-19.	The wearable devices in consumer has some null values as without detecting any values when they not wearing watches during night time, so this study also evaluated all footsteps of consumers. For eliminating this.	Overlapping and missing values are detected.
The biomedical signals are decayed using the TF (time frequency) methods which detect changes in frequency and time^35^	The information and distinctive parameters describes the attitude of signal waveform with accurate action.	It is most important to handle small number of values which characterize the proper features of signals for accomplishing best performance.	It handles only small number of values.
A new facial mask condition identifies methods by combination of pictures by SRCNet (Super resolution and classification networks), calculates three classification problems on unrestrained 2D facial images^49^	The proposed algorithm includes four steps: facial detection, and cropping, image pre-processing, facemask wearing identification and image super resolution.	SRCNet attained 98.70% of accuracy and performs end to end classification methods using DL techniques with super resolution of images over 1.5%.	Future study is to extract real time cough sounds.
Identifies different coughs sounds for altering real time life environments^51^	The first step is transforming stage where sound is converted into an image which is optimized by scalogram tool.The second step includes classification and feature extraction based on deep transferring models such as ResNet50, GoogleNet, ResNet18, MobileNetv2, NasNetmobile and ResNet101	ResNet18 has highest stability for classifying sounds with the sensitive rate of 94.44% and specificity about 95.37%.	Almost all algorithms attained most accurate results. No limitation of this study is presented.
A new method is initiated by integrating the speech and audio signals processing and AI neural networks models using MATLAB software's^40^	A system was developed and designed for identifying the sounds due to collision of hazelnut in steel disk, Microphone was taken under and sounds and noises are recorded in PC through sound card.	Total data signals are divided as 70% data signals are used for training, 15% for validating and remaining are sued for testing AI neural networks	This system is only developed in MSTLAB software.
Analysis is made through the crypt, iris pigment spot and wolflin nodules^41^	The SURF, BRISK, MinEigen, FAST, Harris and MSER are elicited form crypt, pigment spot of iris region and wolflin nodule.	The total inputs are divided into trained and untrained category as 60% and 40%. The authenticated threshold level of crypt, wolflin nodule and pigment spot are as 2, 0, and 3 respectively.	There is many exiting system attains more accuracy than this proposed system.
DWT (Discrete Wavelet transforms), MFCC (Mel Frequency Cepstral Coefficients), ZCR (Zero crossing rate), pitch and energy are used for extracting the features^43^	The work emphasising the pre-process of related audio samples where noises from speech samples has been removed using filters.	In feature selecting stages GFA (Global feature algorithm) is used for removing unwanted details from features and for identifying the emotions form the ML methods. These algorithms validating global emotions like sad, happiness, anger and neutral.	This proposed system monitors all types of emotions using ML methods, sometimes null values may occurs when it exists no emotions.
Developing a new toolbox called SPAC for simulating and extracting speech attributes^44^	The vibrated signals are disintegrated into IMFs (Intrinsic mode functions) by CEEMD algorithm because it has good adaption for extracting non stable signals form features. Then, it improved as LDWPSO algorithm which is initiated for solving the problem where the selection of smooth factor in PNN.	Diagnosing COVID-19 using LDWPSO-PNN.	If speech attributes are known to algorithm it elicited with missing values.

**Fig. 2:**
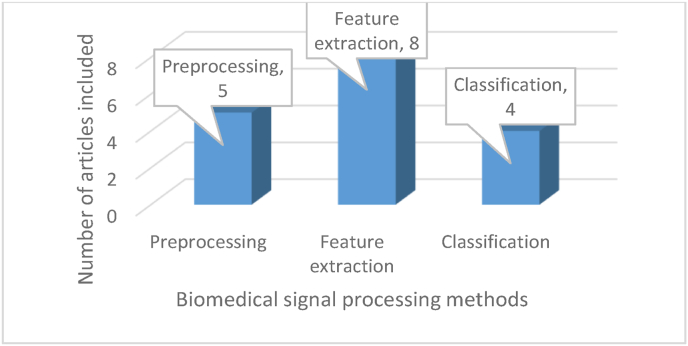
**Number of articles included for biomedical signal processing methods**.

New models takes two steps for determining images: the first steps is transforming stage where sound are converted into images which is optimized by scalogram tool [53]. The second step includes classification and feature extraction based on deep transferring models such as ResNet50, GoogleNet, ResNet18, MobileNetv2, NasNetmobile and ResNet101 [54]. The result of these model shows that ResNet18 has highest stability for classifying sounds with the sensitive rate of 94.44% and specificity about 95.37%. Comparison of this research are made with analysis, hence, this model existed with better accuracy and specificity values. Cough research is precision and more stable for testing generalization and extrapolation [55]. A new method is initiated by integrating the speech and audio signals processing and AI neural networks models. A system was developed and designed for identifying the sounds due to collision of hazelnut in steel disk, microphone was taken under and sounds and noises are recorded in PC through sound card. Then [56], the sounds are further processed and developed in MATLAB software's. A piezoelectric circuit and sensor are used for eliminating ambient noises. The wavelet and time domain data features are extracted using MATLAB and analysed using AI neural network. The total data signals are divided as 70% data signals are used for training, 15% for validating and remaining are sued for testing AI neural networks [57,58]. This Fig. 2 represents number of articles on feature extraction, pre-processing and classification.

## 5 Existing work on wearable devices for tracking COVID 19 symptoms

This study [58] used for identifying SARS-CoV-2 virus which causes respiratory syndrome, it disturbs regular breathing and leading to cough continuously. Automatic respiration monitoring systems provides warnings for timely driven intervention, particularly those with mild type of respiratory problems are used for identifying the symptoms. The present respiratory detecting system costs high so, this easy driven approach used for detection of respiratory problem in lungs [55]. The proposed method used low cost universal ambient sensor and develop a novel signal processing algorithm with Sparsity filter for eliminating frequency noises. Three modes like breathing, coughing and others are detected and estimating its results with accuracy rate of 97.33% and 98.98% of specificity respiration rate. This system is efficient for detecting and screening COVIUD_19 patients with symptoms and also for large scale of patient monitoring systems. Similarly [60], also used for identifying SARS-CoV-2 virus and monitoring RR (respiration rates) of patients who are COVID-19 positive using regular home WiFi. This proposed system suggested Wi-COVID, a non-wearable and non-invasive technology for monitoring patients and tracking RR for the health care provider. A frame work is created for end to end application for monitoring non-invasive platform for COVID-19 patients. A regular WiFi used for making framework platform where patients are monitored in home itself. Another study with wearable physiological signals used for detecting COVID-19 [61]. This work proposes an integrated IoT framework which provides wireless based communication of physiological signals for hub data processing towards LSTM (Long Short Term Memory) based emotions recognition is performed. The existed frame work enables practical communication and recognition of human emotions for monitoring patients with distance learning supports. The proposed system suggested IoT protocols, R-MAC and TS-MAC and ultra-low level latency with 1 Ms is attained. R-MAC provides reliable comparison. This study attained best DL results with the accuracy of F-score 95% [62].

**Fig. 3:**
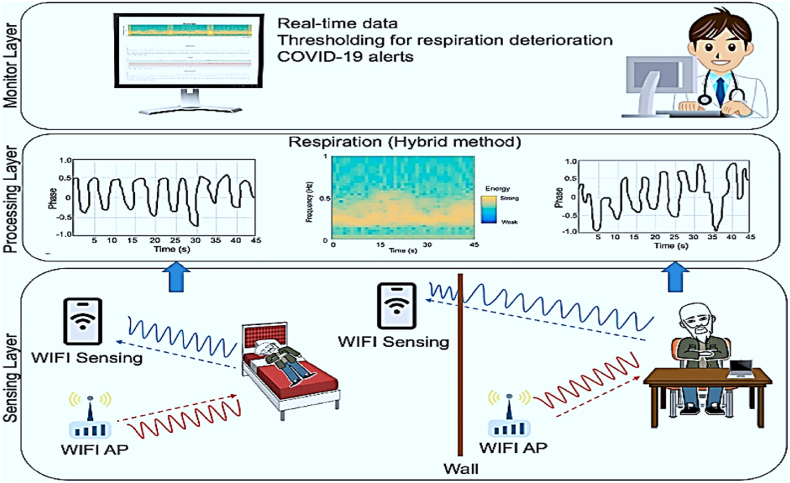
Wi-COVID-19 framework [59].

This above Fig. 3 shows about the frame work of Wi-COVID with three layers. The sensing layers provides commercial device layers of Raspberry PI and estimating RR form CSI signals. In processing layers, the sensed RR are converted into data processing signals using hybrid method. The results are shown to doctor in monitoring layer. Using this data, doctor medicates patients and alters medical staff nearby them [63].

## 6 Photopletysmography signal analysis for biomedical applications

Photoplethysmography is also known as PPG, is a simple and optical technical tool used for detecting volumetrically changes of peripheral circulation in blood. It is a non-invasive and low cost method which forms measurements at the skin's surface. This technique offers sensitive information connected to cardiovascular system. Recent advanced technology is reviving interest in PPG technique and extensively used in clinical physiological monitoring and measuring. So, this optical technique has applied for monitoring HRV and this adopting method has been emerging in medical field when compared to common method, ECG (Electrocardiography). HRV (heart rate variability) has been a significant tool for analysing physiological conditions of patient's as well as aiding a diagnosis method for cardiopathies. Photoplethysmography sensors evaluate the quantity of infrared light reflected or absorbed by blood. If pressure of blood vessels changes, simultaneously volume also changes, which has been arises throughout the cardiac cycle. The function of photoplethysmography has been classified into two types, reflection or transmission of light over or by a specific part of the body. Here [64], PPG technical tool has been used for respiratory and heart rate acquisition, instead of using other technique like ECG. As a result, the safest extraction of respiratory data is retrieved through PPG waveforms, which evaluates values better than ECG signal. PPG has been implemented for both prediction and detection of various diseases, since COVID-19 viruses are also detected by monitoring heart rate of COVID patients and intimated with PPG sensors. Hence, PPG approach has been used for monitoring and measuring of HRV has been detected [65].

### 6.1 PPG monitoring system for detecting COVID-19

As mentioned earlier, PPG signal processing technique used for detecting heart rate of COVID patients. Hence, this has been deeply analysed PPG signals for identifying heart beat rate with PPG dropouts [66]. PPG dropouts are referred as a binary metric where any single heartbeat or peak to peak pulse wave of PPG signal voltage value has been reduced; this is below the PPG dropout threshold. The PPG dropout threshold was shown as the 60 s shifting average of PPG signal minus to 60 s shifting S.D of PPG signal [67]. Each pulse wave had a status whether a dropout or not depended to condition which had been needed. While using PPG pulse rate and dropouts, a ratio of PPG dropouts or dropouts per pulse was obtained as the ratio of amount of dropouts at 1 min divided by pulse rate waves at 1 min [68].

## 7 Challenges

Due to the usability of Artificial intelligence, it is very effective for making our lives smart and easier. The challenges of AI facing, while implementing in various applications forms, here challenges of AI to detect COVID-19 signal processing and applications [69,70]. Challenges faced by AI in health care industry:

• Dividing data for various purposes
• Removing duplicates and errors through reviewing
• AI model as pre-trained application [70].

The challenges while using wearable devices for predicting COVID-19 are: peoples with various mind-set which affects physiological systems, the amplitude of daily activity rhythms, taking suggestion which is displayed among the manuscript, correlation over variables, stability over days, stability of the correlation will change individuals [71]. The data form large population will affects the privacy among democracy and it shares information with various components of COVID-19 patterns. In addition to, the quality of data is determined by software and hardware of large database system includes comfort, ease of user compliance etc. [72] here, first half of data are not used due to the user choices. More cases will fail to generate data because of not wearing that wearable devices, providing symptoms without wearing that wearable devices and failed to communicate some crucial information such as contact details or account information for identifying the data was impossible [73,74].

## 8 Applications

In the emerging technological world, AI transforms health care system with [70].

• AI used for immediate diagnosing.
• AI quickens the Pharma firm's development.
• Health insurance field is automated by AI.
• AI offers health care business [75].

**Some real time examples of AI applications in health care system** [76]**:**

• Robotic surgery
• Detecting COVID-19
• Identifying drug through AI techniques
• Eye surgery by robot
• AI supports admin tasks
• AI image analysing is utilized by health care professional
• Virtually created nursing assistants
• Actions are insights [77].

The adaptation of AI can build education and trust:

There are many countless diseases and health related issues are existed. The basic AI adoption in public department is APM (Alternative Payment Methods), these method are now widely used instead of cash transaction. People trust these alternative methods and slowly insisted other to use APM. In health care system, the advanced technology like AI, ML and DL etc. used for solving data interoperability over this health care system diversely [79]. While implementation of AI in health care system creates awareness and education among people to use AI methods in operation [78].

• APM or (“Alternative Payment Models”)
• Interoperability

While implementing AI for detecting COVID-19 process, it observed 98.9% of accurate values and detects correct symptoms when comparing manually [80]. These values help patients who are in self-quarantine and self-isolation instead of waiting in hospitals. This system serves medical industry during the pandemic situation [81].

## 9 Analysis and discussion

**Fig. 4:**
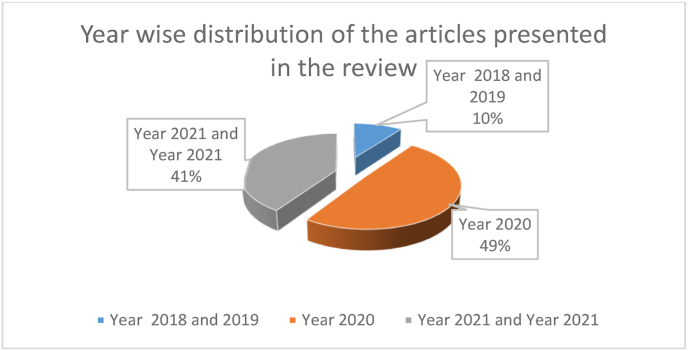
The year wise representation of the article presented in this review.

Fig. 4 represents the year wise distribution of the articles presented in the review. Most of the articles belong to the year 2020 ad 2021 that emphasize that the review has been based on the recent information.

This study examines the patterns of research papers published in COVID-19 on Artificial Intelligence, Machine Learning, and Deep Learning. The majority of the articles were published in the fields of computer science artificial intelligence, computer science information systems, and interdisciplinary sciences. Researchers from all over the world have been trying to focus on COVID19 research and to solve the situation through Artificial Intelligence, Machine Learning, and Deep Learning as the number of COVID-19 cases has increased rapidly and it has appeared as a severe global pandemic. Their main goals are to create and verify new models for efficiently diagnosing, detecting, and stratifying COVID-19 patients. Furthermore, assessing epidemic trends, identifying biomarkers, discovering new treatments, and predicting mortality risk are also topics of interest. Researchers are concentrating their efforts on image analysis research in order to more precisely and quickly screen COVID-19 patients. Almost all of the journals publishing AI, Machine Learning, and Deep Learning research were in the health field, with a heavier emphasis on computer science, artificial intelligence, and computer science information systems, as expected. Artificial Intelligence, Machine Learning, and Deep Learning are gaining popularity in the healthcare industry because of their capacity to solve complicated illness patterns, identify risk earlier, and provide individualised treatment. Artificial Intelligence, Machine Learning, and Deep Learning based technology have been used as a powerful solution for addressing the COVID-19 pandemic, as an AI-based model can handle a large amount of patient data and recognize patterns. While searching for areas of research interest, most of the authors focused on COVID-19 diagnosis, detection, and classification.

## 10 Limitations

Some limitations existed in our investigation. To begin, we included only English-language studies. We may have missed numerous works on Artificial Intelligence, Machine Learning, Deep Learning, and COVID-19 because they were not peer-reviewed. A subsequent study will incorporate PubMed data**.**

## 11 Conclusion

This study provided a detailed summary of current research trends in Artificial Intelligence, Machine Learning, and Deep Learning for COVID-19. This study's findings also revealed that Artificial Intelligence, Machine Learning, and Deep Learning research focus on COVID-19 diagnosis, detection, epidemic patterns, categorization, and medication repurposing. COVID-19 has increased dramatically in the last two years, and early detection is critical for government and public safety. Early diagnosis of this condition requires time and money from the medical and health care companies. To detect COVID-19 at early stages, several researchers use Artificial Intelligence, Machine Learning, and Deep Learning approaches, even at home. As AI, Machine Learning, and Deep Learning become more widely used in clinical practise, which will help to deal with COVID-19 and other pandemics.

## Ethical approval

The Authors declare that no ethical approval is required because research studies don't involve any experiments on patients.

## Sources of funding

The authors declare that of no source of funding for this research studies.

## Author contribution

Formal analysis and writing the manuscript were done by Satyajit Anand and Vikrant Sharma. This work was edited by Rajeev Pourush and Sandeep Jaiswal.

## Consent

The Authors declare that no consent is required because research studies don't involve any experiments on patients.

## Registration of research studies

1. Name of the registry: Not Applicable.

2. Unique Identifying number or registration ID: Not Applicable.

3. Hyperlink to your specific registration (must be publicly accessible and will be checked): Not Applicable.

## Guarantor

Dr Satyajit Anand.

## Funding

There is no funding available for the preparation of manuscript.

## Provenance and peer review

Not commissioned, externally peer reviewed.

## Declaration of competing interest

The authors declare no conflicts of interest.
